# Anatomy of the fetal membranes: insights from spinning disk confocal microscopy

**DOI:** 10.1007/s00404-023-07070-0

**Published:** 2023-05-15

**Authors:** Hannah Marie Eichholz, Alissa Cornelis, Benjamin Wolf, Hanna Grubitzsch, Philip Friedrich, Ahmad Makky, Bahriye Aktas, Josef Alfons Käs, Holger Stepan

**Affiliations:** 1 Leipzig Institute for Meteorology, Leipzig University, 04103 Leipzig, Germany; 2 Center for Scalable Data Analytics and Artificial Intelligence, Leipzig University, 04105 Leipzig, Germany; 3 Department of Obstetrics, University Hospital Leipzig, 04103 Leipzig, Germany; 4 Department of Gynecology, University Hospital Leipzig, 04103 Leipzig, Germany; 5 Peter Debye Institute for Soft Matter Physics, Leipzig University, 04103 Leipzig, Germany; 6 Department of Pathology and Neuropathology, University Hospital and Comprehensive Cancer Center Tübingen, 72076 Tübingen, Germany

**Keywords:** Fetal membranes, Chorion, Amnion, Anatomy, Confocal microscopy, Premature preterm rupture of membranes, Preterm labor, Fluorescence microscopy, Confocal microscopy, Fetal membrane microfractures

## Abstract

**Purpose:**

The fetal membranes are essential for the maintenance of pregnancy, and their integrity until parturition is critical for both fetal and maternal health. Preterm premature rupture of the membranes (pPROM) is known to be an indicator of preterm birth, but the underlying architectural and mechanical changes that lead to fetal membrane failure are not yet fully understood. The aim of this study was to gain new insights into the anatomy of the fetal membrane and to establish a tissue processing and staining protocol suitable for future prospective cohort studies.

**Methods:**

In this proof of principle study, we collected fetal membranes from women undergoing vaginal delivery or cesarean section. Small membrane sections were then fixed, stained for nucleic acids, actin, and collagen using fluorescent probes, and subsequently imaged in three dimensions using a spinning disk confocal microscope.

**Results:**

Four fetal membranes of different types were successfully processed and imaged after establishing a suitable protocol. Cellular and nuclear outlines are clearly visible in all cases, especially in the uppermost membrane layer. Focal membrane (micro) fractures could be identified in several samples.

**Conclusion:**

The presented method proves to be well suited to determine whether and how the occurrence of membrane (micro) fractures and cellular jamming correlate with the timing of membrane rupture and the mode of delivery. In future measurements, this method could be combined with mechanical probing techniques to compare optical and mechanical sample information.

## What does this study add to the clinical work

**Table:** 

Premature and/or preterm fetal membrane rupture is a disastrous tissue failure whose underlying mechanisms are not completely understood. This study demonstrates the great potential of spinning disk confocal microscopy for the investigation of fetal membrane anatomy and provides a tissue processing protocol for future studies.	

## Introduction

The fetal membranes surround the fetus and the amniotic fluid during pregnancy. They are composed of three main layers: (i) the amnion, the innermost epithelial layer surrounding the amniotic fluid, (ii) the chorion connecting the fetal membranes to the maternal decidua, and (iii) a mesenchymal layer between the amnion and chorion containing predominantly collagen-rich extracellular matrix and interspersed amnion mesenchymal cells [1, 2].

The membrane’s integrity until parturition is of vital importance for fetal and maternal health. Preterm birth (PTB)—i.e., delivery earlier than 37 + 0 weeks of pregnancy—is a tremendous public health concern and affects about 15 million babies each year worldwide [3]. PTB manifests initially either as preterm labor or preterm premature rupture of membranes (pPROM). In this process, the fetal membranes are generally regarded as passive bystanders while maternal structures such as the decidua, the myometrium, and the uterine cervix are considered the active players in labor initiation and therefore also the targets of possible intervention [4]. For example, the process of matrix breakdown, membrane separation from the decidua, and ultimately membrane rupture is referred to biochemically as “membrane activation” [4]. This pathway involves the switch from anti-inflammatory to proinflammatory signaling leading to increased oxidative stress and expression of matrix metalloproteinases (among other enzymes) resulting in membrane breakdown [2, 5]. Important research over the past decade has shed more light on the active role of fetal membranes in the initiation of parturition, however. In brief, it has been demonstrated that fetal membrane senescence leads to focal apoptosis and tissue microfractures [6], resulting in the release of damage-associated pattern (DAMP) molecules that lead to a proinflammatory senescence-associated secretory phenotype (SASP) [7–9].

Most research has focused on dynamic biochemical changes and little attention has been paid to constitutional variations of membrane architecture. Whether such inter-individual variants can itself be determinants of membrane breakdown remains to be investigated. We hypothesize that membrane integrity can be conceptualized in anatomical and functional terms (which are necessarily interrelated). While anatomical integrity refers to the structural composition of the membranes as a physical barrier preventing amniotic fluid from leakage and inhibiting the ascent of microorganisms into the amniotic cavity, functional (biochemical) integrity refers to the membranes’ ability to secrete biologically active substances such as prostaglandins, matrix-degrading enzymes, and antimicrobial components. Here we report first an imaging technique suitable for detailed structural analysis of fetal membrane architecture.

## Materials and methods

### Ethics approval, patient selection, and sample collection

This was a single center study, and all specimens were collected at the Perinatal Medicine Department of the Leipzig University Medical Center. All experiments conducted were approved by the local Ethics committee (2021-12-20, 540/21-ek). Women eligible for inclusion were older than 18 years and their babies were delivered at term (37+0 – 41+3 weeks of pregnancy) either vaginally or by cesarean section (see Table 1 for patient characteristics). On admission to the labor and delivery ward, women were asked if they were willing to donate the fetal membranes directly after delivery. After the placenta and fetal membranes were inspected for completeness, the membranes were separated from the placenta and directly transferred to the laboratory at the Peter Debye Institute for Soft Matter Physics in Leipzig for further analysis.

**Table 1:** Fetal and maternal characteristics

Case No.	GA (weeks)	Delivery mode	Number of pregnancies	Number of prev. deliveries	Sex	Birth weight	Additional maternal morbidity
1	38 + 0	VB (induction because of IUGR)	2	1	Male	2700 g	History of HELLP-syndrome in previous pregnancy
2	40 + 3	Cesarean (two previous C-sections)	15	10	Female	3290 g	None
3	39 + 0	Cesarean (maternal tocophobia)	1	0	Male	3610 g	None
4	39 + 4	VB	6	5	Female	3520 g	Insulin-dependent gestational diabetes

### Preparation of the fetal membranes

Upon arrival at the laboratory, the specimens were rinsed with phosphate buffered saline (PBS, Thermo Fisher Scientific, Waltham, Massachusetts) three times to eliminate any fetal contaminations (e.g., meconium stains). A representative part of the membranes measuring approximately 1.5 cm^2^ was excised and fixed with 10% neutral buffered formalin (NBF, Sigma-Aldrich, St. Louis, Missouri) at 4 °C for 24 h.

### Staining

To visualize the cell nuclei, all samples were stained with SPY555-DNA (Spirochrome AG, Stein am Rhein, Switzerland) which is a non-toxic, cell permeable, and highly specific live cell DNA probe that can be excited with wavelengths around 555 nm. In addition, actin and collagen were stained using SiR-actin (Spyrochrome AG, Stein am Rhein, Switzerland) and Col-F Collagen Binding Reagent (ImmunoChemistry Technologies, Davis, California), respectively. The cell permeable SiR-actin probe is highly specific for filamentous actin (F-actin), has an excitation maximum at 652 nm and emits light in the far-red region. Col-F binds to collagen and elastin fibers, which are main components of the extracellular matrix (ECM). The fluorescent probe can be excited at wavelengths around 490 nm and emits green light. Furthermore, 1% of Triton X-100 (Sigma–Aldrich, St. Louis, Missouri) was added to the staining solution to permeabilize the cell membranes and facilitate the uptake of stain molecules.

The tissues were submerged in the staining solution and incubated at 4 °C for 12–24 h. Thereafter, the stained samples were washed with PBS three times and placed in glass bottom Petri dishes (ibidi, Gräfelfing, Germany) with the amnion or chorion facing down. Depending on the size of the tissues, a few drops of ibidi Mounting Medium (ibidi, Gräfelfing, Germany) were added to the samples to reduce photobleaching effects and ensure optimal conditions for microscopic imaging.

### Imaging

Imaging was performed using an inverted Zeiss Axio Observer.Z1 research microscope (Carl Zeiss Microscopy GmbH, Jena, Germany) equipped with a Yokogawa CSU-X1A 5000 spinning disk confocal scanning unit (Yokogawa Denki, Musashino, Japan). This setup enables high-resolution spinning disk confocal microscopy with up to four different wavelengths.

The stained tissue samples were excited with lasers corresponding to their respective stains, namely 488 nm (Col-F), 561 nm (SPY555-DNA), and 638 nm (SiR-actin). They were then imaged for different magnifications and to a depth of about 50 µm from their surface. Subsequently, the individual channels were slightly adjusted in terms of contrast and superimposed.

## Results

**Fig. 1:**
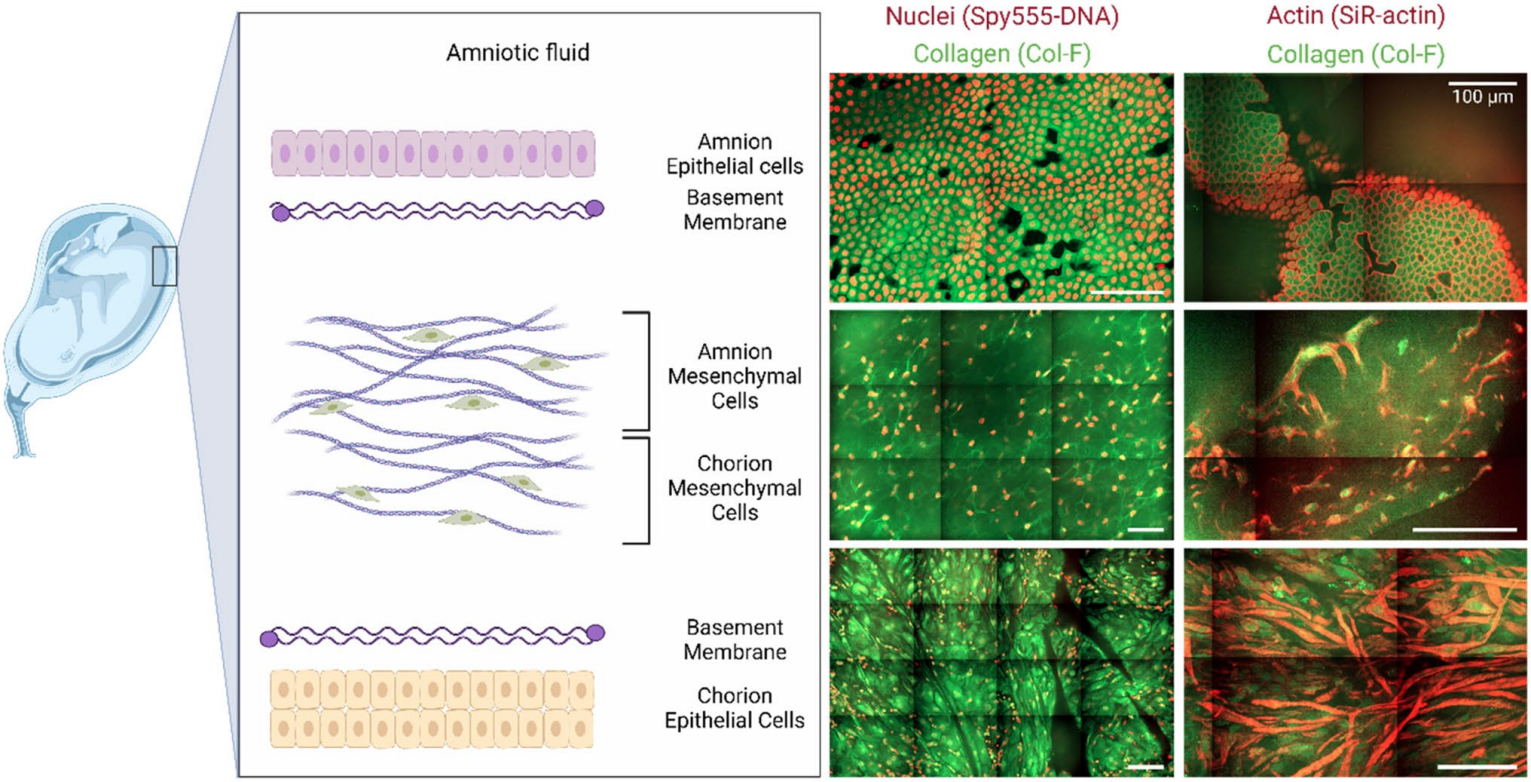
Microscopic fetal membrane anatomy visualized by spinning disk microscopy. On the left, a schematic drawing indicates the relevant anatomical structures. In the middle column, confocal microscopy images with nuclear staining for DNA (red) and collagen (green) are shown. In the right column, corresponding images with staining for actin (red) and collagen (green) are shown

For this proof of principle investigation, four fetal membranes were processed and imaged as described above. The characteristics of the donor women are described in Table 1. Fixation, staining, and imaging of the tissue was successful in all cases, irrespective of delivery mode (vaginal delivery or cesarean section). Image resolution was expectedly best in the most superficial layer (amnion), but sufficient resolution was achieved in the deeper layers of the mesenchymal membrane part as well (compare Fig. 1). Nuclear and cellular outline was excellent on the amniotic surface. In all cases, we could identify focal membrane (micro) fractures as described before [6].

## Discussion

We report here for the first time the utility of spinning disk confocal microscopy for imaging fetal membranes and provide a protocol for tissue processing and staining. A high-resolution, three-dimensional image data acquisition method, as we report here, is the prerequisite for an in-depth investigation of fetal membrane structure. A key advantage of our method is that tissues can be imaged after they have been used for other mechanical studies such as atomic force microscopy (AFM). This will help us to precisely correlate visual and mechanical information in the future.

Richardson et al. have used a combination of multiphoton autofluorescence microscopy and second harmonic generation microscopy to generate three-dimensional images of native fetal membranes [6]. Although this technique provides excellent images, the use of autofluorescence is rather a surrogate than a precise mapping of extracellular matrix components as can be accomplished using specific staining. Furthermore, our images provide a clearer outline of cellular, and more importantly nuclear shapes by actin staining. We have demonstrated before that cell and nuclear shape are a surrogate parameter of cellular unjamming, which is an important determinant of tissue fluidity and therefore stability [10]. It will be interesting to investigate whether cellular unjamming as indicated by cellular and nuclear shapes—which can be analyzed easily—is correlated with time of membrane rupture, pPROM, and delivery mode. We hypothesize that a more fluidic cell shape is associated with pPROM and vaginal birth, while a more static tissue architecture will be observed in patients undergoing cesarean section or iatrogenic membrane rupture during vaginal birth. While a wealth of literature has been published on regional membrane weakening in the supracervical zone [11–14], it will be especially interesting to determine whether architectural changes occur more ubiquitously in cases of (repeated) pPROM. This could indicate a structural predisposition to pPROM, as such membranes are more susceptible to inflammatory attack. Our investigations will, therefore, focus on membrane sections distant from the supracervical region. Bürzle et al. have already reported that a decreased collagen and tissue pyridinoline concentration in fetal membranes correlated with stretch stiffness and membrane tension at rupture in an ex vivo model [15]. Fittingly, in a recent review, Ramkumar Menon and John Moore—two of the most accomplished researchers in the area—have raised the question of how membranes fight off small inflammatory challenges that might otherwise lead to fetal membrane rupture and labor onset. The answer might lie in architectural robustness, just as a well-built fortress will resist heavier attacks. The methods presented in this paper will help us to answer this question.

## Data Availability

This study entails only limited data in the form of microscopy images. Images can be obtained from the corresponding author upon request.

## References

[1] Menon R, Richardson LS, Lappas M (2019). Fetal membrane architecture, aging and inflammation in pregnancy and parturition. Placenta.

[2] Bryant-Greenwood GD (1998). The extracellular matrix of the human fetal membranes: structure and function. Placenta.

[3] Walani SR (2020). Global burden of preterm birth. Int J Gynecol Obstet.

[4] Romero R, Dey SK, Fisher SJ (2014). Preterm labor: one syndrome, many causes. Science.

[5] Strauss Jerome F (2013). Extracellular matrix dynamics and fetal membrane rupture. Reprod Sci.

[6] Richardson LS, Vargas G, Brown T (2017). Discovery and characterization of human amniochorionic membrane microfractures. Am J Pathol.

[7] Dutta EH, Behnia F, Boldogh I (2016). Oxidative stress damage-associated molecular signaling pathways differentiate spontaneous preterm birth and preterm premature rupture of the membranes. Mol Hum Reprod.

[8] Behnia F, Taylor BD, Woodson M (2015). Chorioamniotic membrane senescence: a signal for parturition?. Am J Obstet Gynecol.

[9] Menon R, Moore JJ (2020). Fetal membranes, not a mere appendage of the placenta, but a critical part of the fetal-maternal interface controlling parturition. Obstet Gynecol Clin.

[10] Grosser S, Lippoldt J, Oswald L (2021). Cell and nucleus shape as an indicator of tissue fluidity in carcinoma. Phys Rev X.

[11] Malak TM, Bell SC (1994). Structural characteristics of term human fetal membranes: a novel zone of extreme morphological alteration within the rupture site. Br J Obstet Gynaecol.

[12] Reti NG, Lappas M, Riley C (2007). Why do membranes rupture at term? Evidence of increased cellular apoptosis in the supracervical fetal membranes. Am J Obstet Gynecol.

[13] El Khwad M, Stetzer B, Moore RM (2005). Term human fetal membranes have a weak zone overlying the lower uterine pole and cervix before onset of labor. Biol Reprod.

[14] Mauri A, Perrini M, Mateos JM (2013). Second harmonic generation microscopy of fetal membranes under deformation: normal and altered morphology. Placenta.

[15] Buerzle W, Haller CM, Jabareen M (2013). Multiaxial mechanical behavior of human fetal membranes and its relationship to microstructure. Biomech Model Mechanobiol.

